# Supplementary material for: Plasmacytoid dendritic cell sensing of hepatitis E virus is shaped by both viral and host factors
Source: Life Sci Alliance. 2025 Apr 2;8(6):e202503256. doi: 10.26508/lsa.202503256 (PMC11966012; doi:10.26508/lsa.202503256)
Supplement: Supplementary file 1 [file LSA-2025-03256_TableS1.docx]

**Supplementary Table**

**Supplementary Table 1. Primers used for RT-qPCR**

| Name | Forward/Reverse | Specificity | Sequence 5’-3’ |
| --- | --- | --- | --- |
| HEV | Forward | HEV | GGT GGT TTC TGG GGT GAC |
|  | Reverse | HEV | AGG GGT TGG TTG GAT GAA |
| GAPDH | Forward | human | AGGTGAAGGTCGGAGTCAACG |
|  | Reverse | human | TGGAAGATGGTGATGGGATTTC |
| Interferon lambda 1 IL29 | Forward | human | TCCTAGACCAGCCCCTTCA |
|  | Reverse | human | GTGGGCTGAGGCTGGATA |
| ISG15 | Forward | human | GACAAATGCGACGAACCTCT |
|  | Reverse | human | CGGCCCTTGTTATTCCTCA |
| ISG56 | Forward | human | GGGCAGACTGGCAGAAG |
|  | Reverse | human | CTATAGCGGAAGGGATTTGA |
| MXA | Forward | human | ACAGGACCATCGGAATCTTG |
|  | Reverse | human | CCCTTCTTCAGGTGGAACAC |
| TNF alpha | Forward | human | AGATGATCTGACTGCCTGGG |
|  | Reverse | human | CTGCTGCACTTTGGAGTGAT |
| IL6 | Forward | human | GTCAGGGGTGGTTATTGCAC |
|  | Reverse | human | AGTGAGGAACAAGCCAGAGC |
| OAS2 | Forward | human | CCTGAAGCCCTACGAAGA |
|  | Reverse | human | TTAAGACTGTTTTCCGTCCA |
